# Premature senescence of endothelial cells upon chronic exposure to TNFα can be prevented by N-acetyl cysteine and plumericin

**DOI:** 10.1038/srep39501

**Published:** 2017-01-03

**Authors:** Shafaat Y. Khan, Ezzat M. Awad, Andre Oszwald, Manuel Mayr, Xiaoke Yin, Birgit Waltenberger, Hermann Stuppner, Markus Lipovac, Pavel Uhrin, Johannes M. Breuss

**Affiliations:** 1 Department of Vascular Biology and Thrombosis Research, Center for Physiology and Pharmacology, Medical University of Vienna, 1090 Vienna, Austria; 2 Department of Zoology, University of Sargodha, 40100 Sargodha Pakistan; 3 King’s British Heart Foundation Centre, King’s College London, London SE5 9NU, UK; 4 Institute of Pharmacy/Pharmacognosy and Center for Molecular Biosciences Innsbruck (CMBI), University of Innsbruck, 6020 Innsbruck, Austria; 5 Karl Landsteiner Institute for Cell-based Therapy in Gynecology, 2100 Korneuburg, Austria

## Abstract

Cellular senescence is characterized by a permanent cell-cycle arrest and a pro-inflammatory secretory phenotype, and can be induced by a variety of stimuli, including ionizing radiation, oxidative stress, and inflammation. In endothelial cells, this phenomenon might contribute to vascular disease. Plasma levels of the inflammatory cytokine tumor necrosis factor alpha (TNFα) are increased in age-related and chronic conditions such as atherosclerosis, rheumatoid arthritis, psoriasis, and Crohn’s disease. Although TNFα is a known activator of the central inflammatory mediator NF-κB, and can induce the intracellular generation of reactive oxygen species (ROS), the question whether TNFα can induce senescence has not been answered conclusively. Here, we investigated the effect of prolonged TNFα exposure on the fate of endothelial cells and found that such treatment induced premature senescence. Induction of endothelial senescence was prevented by the anti-oxidant N-acetyl cysteine, as well as by plumericin and PHA-408, inhibitors of the NF-κB pathway. Our results indicated that prolonged TNFα exposure could have detrimental consequences to endothelial cells by causing senescence and, therefore, chronically increased TNFα levels might possibly contribute to the pathology of chronic inflammatory diseases by driving premature endothelial senescence.

Cardiovascular diseases are the leading cause of death in the elderly population of western countries^1^. Endothelial cells form the inner lining of the vasculature and regulate vascular tone and hemostasis, thus playing a pivotal role in vascular function^2^. Evidence indicates that cellular senescence, characterized by a cell-cycle arrest and pro-inflammatory changes in gene expression^3^, occurs in endothelial cells *in vivo* and may play a role in age-related vascular pathology such as atherosclerosis, e.g. by reducing important vasodilatory factors such as nitric oxide and prostacyclin and promoting a pro-adhesive and pro-thrombotic phenotype^3^,^4^,^5^,^6^,^7^,^8^.

Senescence can be induced by a plethora of stimuli, including ionizing radiation^9^,^10^ telomere dysfunction^4^,^11^, reactive oxygen species (ROS)^12^,^13^, high glucose concentrations^14^,^15^ or inflammatory cytokines^16^,^17^. It has been established that the underlying cell-cycle arrest is mediated by p21 and p16, two cyclin-dependent kinase inhibitors^18^,^19^,^20^, and that persistent DNA damage signaling drives the hallmark - inflammatory and tumorigenic - phenotype of senescent cells, termed the senescence-associated secretory phenotype (SASP)^21^,^22^. This SASP, which prominently involves NF-κB signaling^23^,^24^, comprises adhesion molecules, metalloproteinases, and many cytokines^3^,^25^,^26^,^27^. Some of these, such as IL-1, IL-6, and TNFα, have been implicated in atherosclerosis^28^,^29^ and diabetes^30^.

Although TNFα is a known activator of NF-κB, and can induce the intracellular generation of ROS^31^, the question whether prolonged exposure to TNFα can induce senescence in endothelial cells has not been answered. Since many SASP genes are responsive to TNFα stimulation within a short time and play an essential role in acute inflammation^32^, it could be important to discriminate between short- and long-term effects of TNFα on endothelial senescence.

In the present study, we investigated whether prolonged stimulation with TNFα might induce a senescence phenotype in human umbilical vein endothelial cells (HUVECs) *in vitro*. We addressed this by assessing the proliferative marker Ki-67, the cyclin-dependent kinase inhibitors p16 and p21, as well as components of the aforementioned SASP, namely E-selectin, intracellular adhesion molecule-1 (ICAM-1), plasminogen activator inhibitor-1 (PAI-1), insulin like growth factor binding protein 5 (IGFBP-5) as well as the cytokines IL-6 and IL-8. In addition, we examined the involvement of NF-κB activity and ROS generation in this process, by assessing nuclear levels of the p65 NF-κB subunit, and employing the commercially available ROS probe H2-DCF. Furthermore, we studied the effect of two IKK2- targeting inhibitors of NF-κB signaling - the synthetic PHA-408^33^ and the plant-derived plumericin^34^ - as well as the anti-oxidant N-acetyl cysteine (NAC)^35^,^36^, on the induction of senescence features induced by TNFα in HUVECs.

## Results

### Chronic TNFα exposure induces cell-cycle arrest in HUVECs

**Figure 1:**
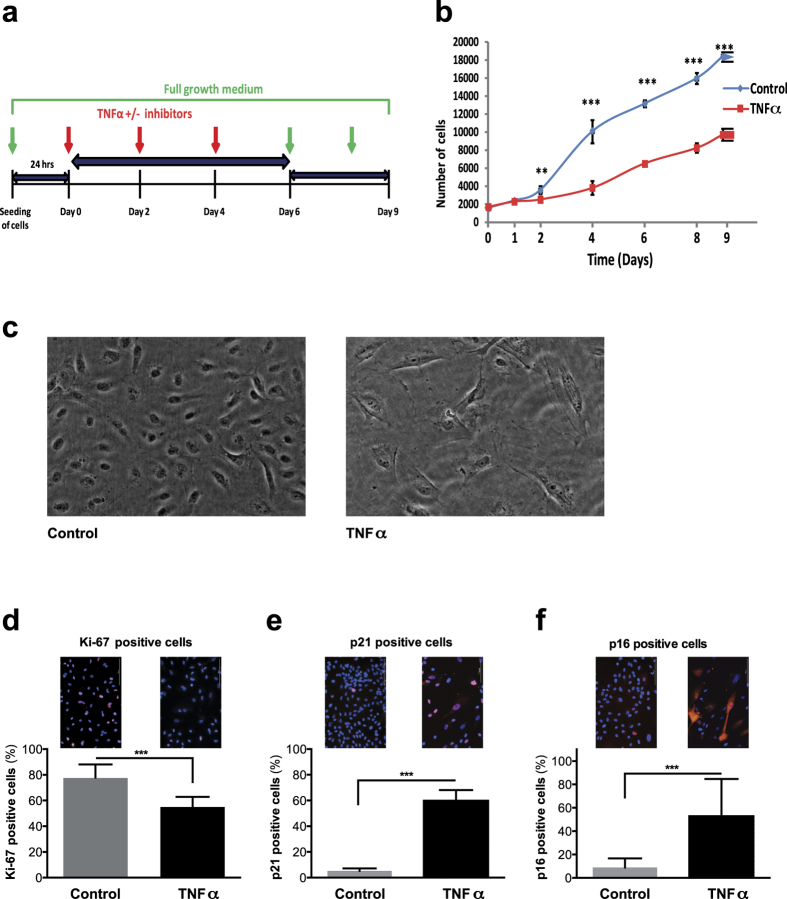
TNFα-induces inhibition of cell proliferation and premature senescence in HUVECs. (**a**) Experimental design: Upon propagation in full growth medium for 24 hours, cells were exposed to TNFα (10 ng/ml) ± inhibitors (NF-κB inhibitors plumericin (1.5 μM), PHA-408 (2 μM) and antioxidant NAC (2 mM)) for a period of six days, followed by a three days recovery period in full growth medium. Control HUVECs were grown in full growth medium only during the whole period. (**b**) Growth curves of HUVECs treated with or without TNFα for the indicated time points. (**c**) Increase in size and flattening of TNFα-treated cells. (**d–f**) Representative fluorescent images of HUVECs (day nine) grown in presence or absence of TNFα and their quantification: (**d**) Ki-67, (**e**) p21, and (**f**) p16.Values are presented as mean ± SD of technical triplicates (**p < 0.01, ***p < 0.001). The results shown are representative of three independent experiments.

To test the hypothesis that chronic stimulation of endothelial cells with TNFα might induce premature cellular senescence, we exposed HUVECs propagated in full growth medium to 10 ng/ml TNFα for six days. This induction period was followed by an additional recovery period of three days in full growth medium only, in order to determine the persistence of the growth arrest after six days of TNFα stimulation (Fig. 1a). As a control, HUVECs were exposed solely to the solvent (0.01% DMSO). The acquisition of features associated with senescence was tested using published markers, including the proliferation marker Ki-67 and the cyclin-dependent kinase inhibitors p16 and p21. Standard staining controls were applied.

We found that the growth rate of cells brought into contact with TNFα for six days was lower than that of control cells, exposed to full growth medium only (Fig. 1b). Following treatment, we observed an increase in size and flattening of cells, a common feature of senescence (Fig. 1c). Staining of treated and control HUVECs with Ki-67 revealed that only half as many cells (p < 0.001) expressed this proliferation marker at the end of six days of TNFα stimulation (data not shown). After the recovery phase of three days, the percentage of Ki-67 positive cells decreased further in the previously treated cells (Fig. 1d). Staining with p21 or p16 revealed that TNFα treatment of HUVECs yielded significant increases (both p < 0.001) in cyclin-dependent kinase inhibitor expression (Fig. 1e,f) as compared to the controls.

We found a similar increase in p16 positivity in human dermal microvascular endothelial cells (HDMECs) after their exposure to TNFα for six days (Supplemental Fig. 1a). These cells also exhibited an increase in size and cell flattening, similar to TNFα–treated HUVECs (Supplemental Fig. 1b).

Furthermore, we analyzed effects of prolonged cultivation in TNFα (10 ng/ml) in HUVECs that were exposed as previously for six days to TNFα, but cultivated for a longer recovery period (7–10 days) without TNFα. We show that this treatment resulted in progressive retardation of growth rate during the analyzed period (Supplemental Fig. 2a). The growth curve did not, though, reach a complete plateau, likely due to heterogeneity in senescence induction, which left a portion of cells still able to proliferate. Nevertheless, the portion of p16 positive cells was much higher in TNFα-treated cells in comparison to controls, as detected at days 13 and 16 (Supplemental Fig. 2b).

Hence, these results showed a decrease in proliferative capacity in HUVECs after their prolonged TNFα exposure, and we investigated whether this treatment might evoke further features of senescence.

### Chronic TNFα exposure promotes the senescence-associated secretory phenotype

In previous studies, senescence was shown to confer characteristic changes in gene expression, i.e. of inflammatory adhesion molecules, cytokines, and matrix metalloproteinases, collectively referred to as SASP^3^,^25^. To determine if our treatment with TNFα would also induce components of the SASP, we assessed the endothelial adhesion proteins ICAM-1 and E-selectin, the prothrombotic PAI-1, IGFBP-5, as well as two cytokines IL-6 and IL-8, shown previously to be elevated in endothelial cells at replicative senescence^7^,^37^,^38^,^39^,^40^. Levels of ICAM-1, E-selectin, IL-6, and IL-8 in TNFα-treated cells were determined using cell ELISA^41^ and levels of PAI-1 and IGFBP-5 mRNA were measured using qPCR. As a comparison, cells not treated with TNFα were similarly assessed.

**Figure 2:**
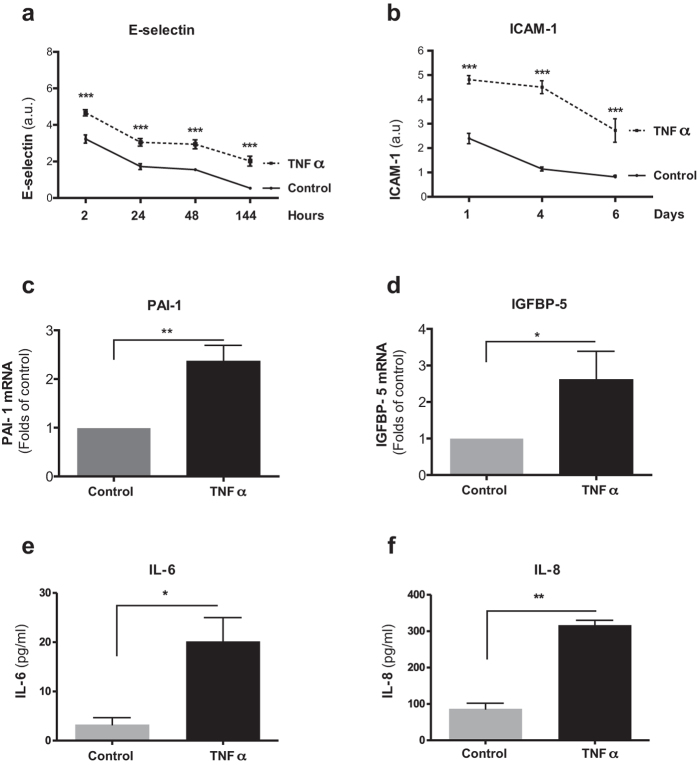
Prolonged TNFα-exposure promotes SASP in HUVECs. (**a**,**b**) Surface expression of E-selectin (**a**) and ICAM-1 (**b**) of HUVECs grown in the presence of TNFα (10 ng/ml) in comparison to control HUVECs for indicated time points, quantified by cell ELISA. (**c**,**d**) mRNA levels of PAI-1 (**c**) and IGFBP-5 (**d)** of HUVECs grown in the presence of TNFα (10 ng/ml) in comparison to control HUVECs at day six, quantified by qPCR. Levels of (**e**) IL-6 and (**f**) IL-8 in supernatants of HUVECs grown in the presence of TNFα (10 ng/ml) in comparison to control HUVECs at day six, quantified by ELISA. Values are presented as mean ± SD of technical triplicates (**a–d**) or technical duplicates (**e,f**). (*p < 0.05, **p < 0.01, ***p < 0.001). a.u.: arbitrary units. The results shown are representative of three independent experiments.

The ELISA results showed that in the cells pretreated with TNFα, adhesion molecules E-selectin and ICAM-1 were significantly increased at all time points compared to control HUVECs (Fig. 2a,b). QPCR analysis at day six demonstrated a statistically significant increase in the abundance of the respective PAI-1 and IGFBP-5 mRNA levels (Fig. 2c,d). Importantly, levels of cytokines IL-6 and IL-8 were significantly increased at day six of TNFα treatment (Fig. 2e,f), pointing to the induction of these cytokines following TNFα exposure. Similarly, levels of IL-6 and IL-8 were also increased in HDMECs that were exposed for six days to TNFα (Supplemental Fig. 1c,d).

In addition, we analyzed IL-6 and IL-8 levels in an experiment of prolonged cultivation in TNFα (10 ng) in HUVECs that were exposed as previously for six days to TNFα, but cultivated further for additional 10 days. The levels of both these cytokines were high, albeit declining, throughout the six-day period of direct TNFα stimulation. In spite of their tendency to further decline, they remained significantly elevated also during the entire 10 days period after withdrawal of TNFα treatment (Supplemental Fig. 2c,d).

Hence, these data demonstrated the induction of several SASP-related proteins upon prolonged TNFα exposure, further supporting the idea that inflammatory stress might promote cellular senescence.

### NF-κB translocation and ROS production are enhanced in TNFα treated cells

Emerging hallmarks in cellular senescence are increased activity of the NF-κB pathway and increased generation of ROS^23^,^42^,^43^. To investigate the time course of NF-κB activation in HUVECs in response to TNFα, we assessed the translocation of the NF-κB subunit p65 over the course of two days using a commercially available antibody and fluorescence microscopy. ROS levels were evaluated based on a fluorescence method applying H2-DCF^44^ and in comparison to control cells.

**Figure 3:**
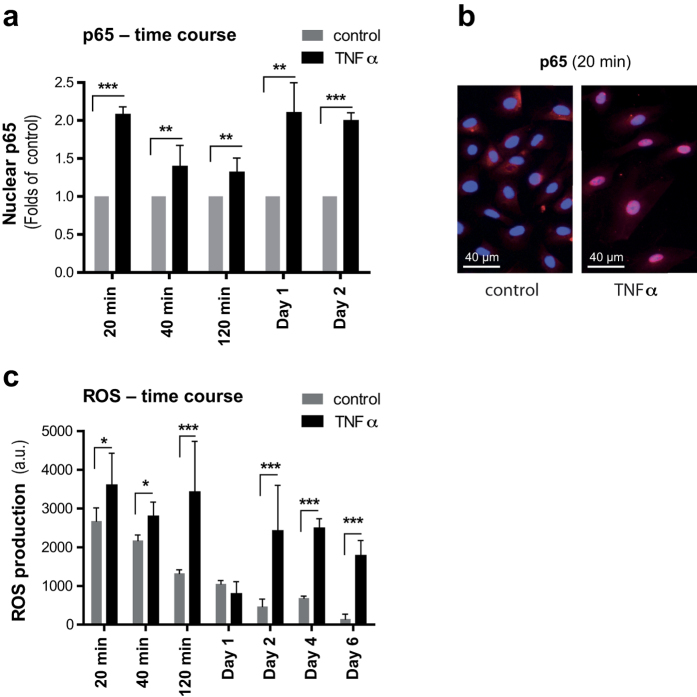
TNFα increases the nuclear translocation of NF-κB and induces ROS generation in HUVECs. (**a**) Quantification of nuclear translocation of NF-κB in HUVECs grown in the presence of TNFα (10 ng/ml) for indicated time points, in comparison to control HUVECs, by integrated mean intensity of p65 in nucleus using CellProfiler software. (**b**) Representative fluorescent images of nuclear translocation of NF-κB in HUVECs (20 min) grown in presence or absence of TNFα. (**c**) Determination of ROS production in HUVECs grown in the presence of TNFα (10 ng/ml) for indicated time points, in comparison to control HUVECs, by using fluorophore H2-DCF (10 μM). Values are presented as mean ± SD of technical triplicates (*p < 0.05, **p < 0.01, ***p < 0.001). a.u.: arbitrary units. The results shown are representative of three independent experiments.

We found a statistically significant increase in the nuclear levels of p65 in TNFα-treated HUVECs at 20 min (Fig. 3a,b) compared to the controls, which were less pronounced but still significant at 40 min and at two hours (Fig. 3a). At day one and day two time points, NF-κB translocation was also significantly increased, and higher than at two hours (Fig. 3a), suggesting a biphasic response. Similarly, ROS production increased over the first two hours, thereafter dropped to basal levels at day one, and was increased again between days two and six (Fig. 3c).

In addition, we analyzed ROS production in an experiment of prolonged cultivation where HUVECs were exposed, as previously, for six days to TNFα, but cultivated further for additional 7 days. We found that ROS levels remained significantly elevated after withdrawal of TNFα treatment (Supplemental Fig. 2e).

Finally, we also investigated if the above regimen of TNFα treatment might induce apoptosis. To this, we used combination of annexin V and propidium iodide staining. The levels of apoptosis were similarly low in control as well as in TNFα-stimulated HUVECs at all analyzed time points (Supplemental Fig. 2f).

Therefore, by demonstrating increased NF-κB translocation and ROS production, as well as showing that TNFα treatment at the used concentration of 10 ng/ml did not induce apoptosis, we could support the notion that prolonged TNFα treatment of HUVECs would cause them to senesce.

### Inhibition of ROS and NF-κB translocation prevents TNFα–induced cell-cycle arrest

We next questioned if the inhibition of NF-κB and/or ROS production would prevent the effects we observed after prolonged TNFα stimulation in HUVECs. We employed inhibitors of NF-κB signaling, including PHA-408^33^ and plumericin^34^ that target IKK2 (a kinase targeting the inhibitors of NF-κB), and other inhibitors that act against ROS, including the anti-oxidant NAC^35^,^36^ and diphenyleneiodonium chloride (DPI), a known inhibitor of NADPH oxidase^45^. We contemporaneously applied TNFα and each of these inhibitors to HUVECs, and measured their effects on translocation of the NF-κB subunit p65 and generation of ROS, using the fluorescent methods described previously^44^. TNFα-non treated HUVECs incubated with inhibitors were considered as controls.

**Figure 4:**
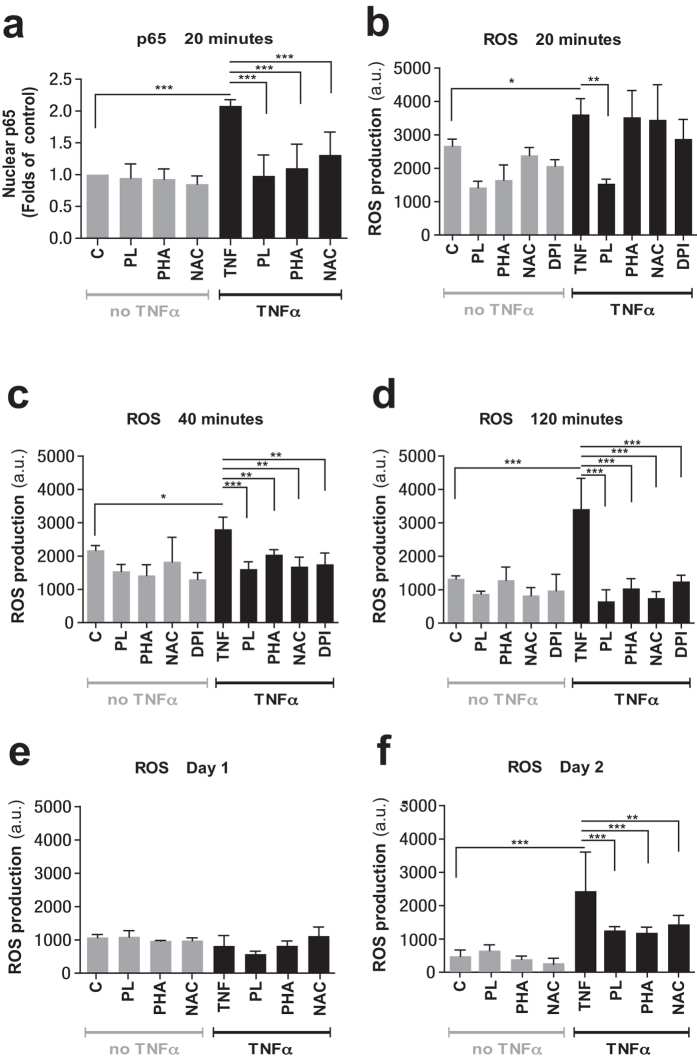
Plumericin, PHA-408, and NAC prevent TNFα-induced nuclear translocation of NF-κB and ROS generation in HUVECs. (**a**) Quantification of nuclear translocation of NF-κB in HUVECs grown in the presence of TNFα (10 ng/ml) ± inhibitors in comparison to control HUVECs ± inhibitors at 20 min, by integrated mean intensity of p65 in nucleus using CellProfiler software. (**b–f**) Determination of ROS production in HUVECs grown in the presence of TNFα (10 ng/ml) ± inhibitors in comparison to control HUVECs ± inhibitors for indicated time points, by using fluorophore H2-DCF (10 μM). Values are presented as mean ± SD of technical triplicates (*p < 0.05, **p < 0.01, ***p < 0.001). The results shown are representative of three independent experiments. C: control, PL: plumericin, PHA: PHA-408, NAC: N-acetyl cysteine, a.u.: arbitrary units.

The results showed that each of the inhibitors attenuated TNFα-induced NF-κB translocation as well as ROS generation (Fig. 4). In the absence of inhibition, TNFα caused an approximately onefold increase in nuclear translocation of p65 of NF-κB (Fig. 4a, column 5). However, when plumericin, PHA-408, or NAC were present, the increased p65 translocation was largely prevented. Application of inhibitors to cells not treated with TNFα (Fig. 4a, columns 2–4) did not alter the nuclear p65 levels. In reference to ROS production, plumericin was able to limit TNFα-induced ROS production to basal levels following 20 min (Fig. 4b, column 7), while the other inhibitors tested took longer to negate ROS production (Fig. 4b–d). The effect of these inhibitors on control cells not treated with TNFα was minor. DPI, when co-applied to the TNFα treated cells, blocked ROS generation, albeit only after 40 min (Fig. 4b–d). In non-treated cells, it did not significantly alter this process.

Since the used inhibitors were efficient in blocking both p65 translocation and ROS production, we further tested if they might also affect features of endothelial senescence.

We first analyzed the ability of these inhibitors to prevent the associated cell-cycle arrest. HUVECs were propagated in full growth medium and treated with 10 ng/ml TNFα for six days in the presence of the aforementioned inhibitors. As before, this induction period was followed by an additional recovery time of three days in full growth medium. HUVECs growth was quantified until day nine, and expression levels of Ki-67, p21, or p16 were assessed by standard immunocytochemistry techniques in combination with commercially available antibodies at day nine. Similar steps were undertaken using control HUVECs that were not pretreated with TNFα.

**Figure 5:**
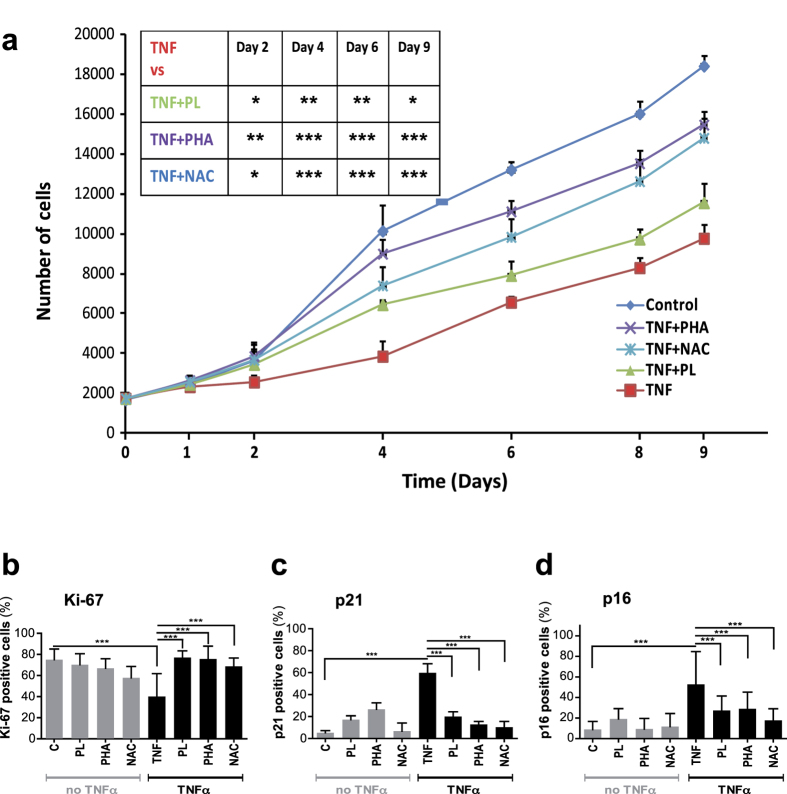
Plumericin, PHA-408, and NAC inhibit TNFα-induced cell-cycle arrest in HUVECs. (**a**) Growth curves of HUVECs treated with TNFα ± inhibitors in comparison to control HUVECs for indicated time points. (**b,d**) Quantification of staining for (**b**) Ki-67, (**c**) p21 and (**d**) p16 of HUVECs treated with TNFα ± inhibitors in comparison to control HUVECs ± inhibitors at day nine. Values are presented as mean ± SD of technical triplicates. (*p < 0.05, **p < 0.01, ***p < 0.001). The results shown are representative of three independent experiments. C: control, PL: plumericin, PHA: PHA-408, NAC: N-acetyl cysteine.

In these control cells, the addition of inhibitors changed growth dynamics only minimally compared to control cells cultured without them (Supplemental Fig. 3a). In contrast, all three inhibitors significantly mitigated the TNFα-induced growth impairment of HUVECs over the whole analyzed period of nine days (Fig. 5a) and attenuated the decrease in proliferating cells, as evidenced by immunofluorescent detection of Ki-67, as well as the increase in cells positive for p21 and p16 (Fig. 5b–d). These data suggest that NAC, plumericin, and PHA-408 were able to prevent TNFα-induced cell-cycle arrest. Therefore, as further evidence for the prevention of senescence, we intended to evaluate further the phenotype of these cells.

### Inhibition of ROS and NF-κB signaling prevents induction of SASP

Having shown that inhibitors of ROS and NF-κB attenuated the induction of a cell cycle arrest by TNFα, we next wished to examine whether their use would also affect the induction of SASP. To this end, we assessed the expression of six endothelial SASP proteins (E-selectin, ICAM-1, IGFBP-5, PAI-1, IL-6, and IL-8) upon utilization of our inhibitors. Untreated HUVECs were used as controls.

**Figure 6:**
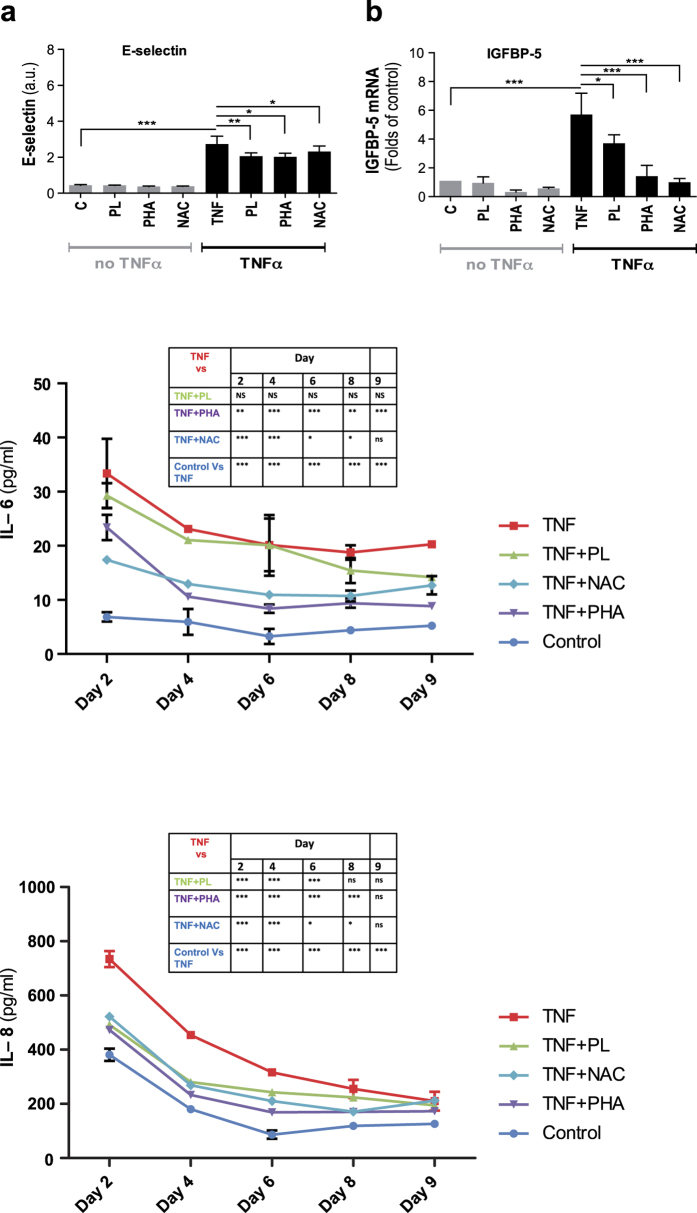
Plumericin, PHA-408, and NAC prevent induction of SASP in HUVECs. (**a**) Surface expression of E-selectin quantified by cell ELISA at day nine and (**b**) mRNA level of IGFBP-5 quantified by qPCR of HUVECs grown in the presence of TNFα (10 ng/ml) ± inhibitors in comparison to control HUVECs ± inhibitors. (**c**) IL-6 levels and (**d**) IL-8 levels in HUVECs grown in the presence of TNFα (10 ng/ml) ± inhibitors in comparison to control HUVECs. Values are presented as mean ± SD of technical triplicates (**a,b**) or duplicates (**c,d**). (*p < 0.05, **p < 0.01, ***p < 0.001). The results shown are representative of three independent experiments. C: control, PL: plumericin, PHA: PHA-408, NAC: N-acetyl cysteine.

The results showed that addition of the three inhibitors (NAC, plumericin, and PHA-408) on their own hardly affected E-selectin levels (Fig. 6a). However, TNFα treatment led to an approximately fivefold higher level of E-selectin, but this increase was reduced by about 20% when the three inhibitors had been applied (Fig. 6a). The results for IGFBP-5 yielded essentially the same pattern (Fig. 6b). Investigation of the expression of ICAM-1 and PAI-1 showed less marked changes (not shown). Importantly, levels of the cytokines IL-6 and IL-8, which were significantly increased upon prolonged TNFα exposure, were reduced by employing the above inhibitors, as determined by analyzing conditioned media (Fig. 6c,d). The levels of IL-6 and IL-8 in the presence of inhibitors and in control cells are presented in Supplemental Fig. 3b,c.

Hence, taking into account a representative cell surface protein (E-selectin) and the secreted factors IGFBP-5, IL-6 and IL-8, we were able to underscore that the prevention of the growth arrest coincided with a reduction of the senescence associated secretory phenotype in HUVECs.

## Discussion

Senescence of endothelial cells, encompassing an irreversible growth arrest and secretion of pro-inflammatory mediators, may contribute to age-related endothelial dysfunction and vascular pathology^6^. In the present work, we showed that long-term application of TNFα to HUVECs induced features of senescence, such as a cell-cycle arrest and secretion of selected SASP components. In correspondence with previous studies, our results suggest the involvement of NF-κB signaling and ROS generation in evoking senescence, as both inhibitors PHA-408 and plumericin, as well as the antioxidant NAC, were able to attenuate the induction of features of senescence.

TNFα has been shown previously to induce senescence in certain cell types^46^,^47^, including fibroblasts^16^, but it remained hitherto unclear whether this is also true for endothelial cells, exemplified by HUVECs, a routinely used endothelial culture model. Although it was reported that TNFα may reduce proliferative capacity in these cells^48^, it was not determined if this was due to senescence. A further study demonstrated that this cytokine may induce certain markers of dysfunction and possibly initiate senescence in HUVECs within 20 hours^49^, but it was not investigated if such changes persisted after the end of treatment.

In our study, we found that six days of treatment with TNFα were sufficient to induce features of senescence that persisted after removal of TNFα. Moreover, we found that the concomitant addition of IKK2 inhibitors or antioxidants with TNFα treatment mitigated the TNFα-induced growth impairment. Our data collectively suggest that although certain features of senescence may be observed shortly after treatment with TNFα, prolonged stimulation may promote the transition from a potentially reversible response towards manifestation of senescence.

A characteristic feature of cellular senescence is the SASP^25^,^26^,^27^,^50^ involving increased surface expression and release of inflammatory molecules^51^. In the present work, we chose to assess the expression of the adhesion molecules E-selectin and ICAM-1, as well as of PAI-1, IGFBP-5, IL-6, and IL-8, previously reported to be upregulated in senescence^7^,^37^,^38^,^39^,^40^,^52^,^53^. We found that the expression levels of all of these proteins were elevated after six days of treatment, and with the exception of PAI-1, remained high for at least three further days of cell culturing in normal growth medium, thereby supporting the notion of their senescence. The induction of these changes was attenuated by concomitant application of inhibitors of ROS production and NF-κB during TNFα treatment. In the case of IL-6 and IL-8, we observed a decline of expression over the course of stimulation. However, their levels remained significantly elevated over control cells at all investigated time points. While these findings support the notion of senescence in our cells, they also suggest that the effect of TNFα on the expression of certain cytokines may be greater than that elicited by the induction of senescence.

ROS generation in response to TNFα is known to occur both in the mitochondria and at the plasma membrane^31^,^54^,^55^,^56^,^57^,^58^ and is believed to contribute to endothelial dysfunction, in part by decreasing the availability of vasodilatory nitric oxide^54^. ROS also influence - both positively and negatively, at multiple signaling levels - the transcriptional activity of NF-κB^59^,^60^. We explored the involvement of NF-κB and ROS in our model of TNFα-induced senescence by employing immunocytochemical staining of the NF-κB subunit p65 and utilizing the commercially available ROS probe H2-DCF. We found that both, the antioxidant NAC, as well as the IKK2 inhibitors PHA-408 and plumericin, suppressed the TNFα-induced translocation of p65 to the nucleus at 20 min time-point (Fig. 4a). However, our results indicate that only plumericin blocked the TNFα-induced increase in ROS at this early time point. In addition, this early increase in ROS appeared to be independent of the NADPH oxidase or the mitochondrial NADH-ubiquinone oxidoreductase, which were reported to be susceptible to inhibition by DPI^45^,^61^.

We further found that TNFα-induced ROS generation, although significant already at 20 min, continued to show a relative increase over the next 120 min. At these later time points, all tested inhibitors, including DPI, potently mitigated ROS generation, thus indicating a likely involvement of NADPH oxidase. Although after 24 hours of treatment, levels of ROS in TNFα-treated cells appeared to be similar to untreated and concomitantly inhibited cells, we observed a second stage of increased ROS levels from day two to six. During this stage, our inhibitors appeared to mitigate potently the increase in ROS observed under TNFα stimulation. Intriguingly, the mean nuclear levels of p65 followed a similar biphasic response in TNFα treated cells, although they declined after a first peak at 20 min, and again after a second peak at 24 hours. Such biphasic response under continuous TNFα stimulation has been previously observed and was correlated with transient degradation of IκBα at early time-points and persistent downregulation of IκBα at late time points^62^. We observed, at the (early) 20 min and 40 min time points, elevated ROS levels also in control cells, possibly due to cell culture stress and persisting effects of harvesting and re-seeding.

Plumericin was originally identified as an inhibitor of IKK2^34^, but has recently been demonstrated to also deplete glutathione levels in smooth muscle cells, presumably due to its high thiol reactivity^63^. Depletion of glutathione has been shown to augment TNFα-induced ROS production in HUVECs^64^,^65^. However, our results indicate that the overall effect of plumericin on ROS production is suppressive. Collectively, the results indicate that plumericin, while having a more pronounced effect on ROS production (especially at early time points), had a lesser effect on rescuing TNFα-impaired cell proliferation and preventing expression of SASP proteins than the specific NF-κB inhibitor PHA-408 or the ROS inhibitor NAC.

In conclusion, we showed that prolonged stimulation of HUVECs by TNFα induced a senescent phenotype. Therefore, premature endothelial senescence may contribute to the pathology of chronic inflammatory diseases in which TNFα is presumed to play a critical role. We believe that our findings may motivate other researchers to explore in more detail the roles of TNFα and endothelial senescence in chronic disease and aging.

## Materials and Methods

### Chemicals and reagents

Plumericin was isolated from bark material of *Himatanthus sucuuba*, as described earlier^66^. PHA-408 was obtained from Axon Medchem BV (Groningen, Netherlands) and NAC, DPI, Triton X-100, medium M199, fetal bovine serum (FBS), goat serum, gelatin, and paraformaldehyde (PFA) were purchased from Sigma Aldrich (Saint Louis, USA). Penicillin, streptomycin, fungizone, and trypsin-EDTA were bought from LONZA (Visp, Switzerland), endothelial cell growth supplement (ECGS) with heparin from PromoCell (Heidelberg, Germany), and human recombinant TNFα from PeproTech (Vienna, Austria). The products 2′,7′-dichlorodihydrofluresceindiacetate (H2-DCF) and TMB ELISA substrate were obtained from ThermoFischer Scientific (Vienna, Austria). Hoechst 33342 and Alexa 555 were obtained from Life Technologies (Columbus, USA), mouse IgG HRP-linked antibody from GE Health care (Little Chalfont, UK), anti-CD31 and anti-p16 from BioLegend (San Diego, USA), anti-p65 from Santa Cruz (Santa Cruz, USA), anti-Ki-67 from ThermoFischer Scientific, anti-p21 from BD Bioscience (Franklin Lakes, USA), anti-E selectin and anti-ICAM-1 from R&D Systems (Abingdon, UK). Trizol, MuLV-reverse transcriptase, RNAse inhibitor and, oligo dT primers were obtained from Life Technologies, and FastStart SYBR Green Master Mix from ThermoFischer Scientific.

### Isolation and culturing of primary endothelial cells

HUVECs were isolated from umbilical cords as described earlier^67^. Subconfluent cultures of HUVECs were cultured at 37 °C in a CO_2_ incubator with 95% humidity in M199 medium containing 20% FBS, supplemented with 3 mg/ml ECGS and 22.5 mg/ml heparin, 2 mM L-glutamine, 100 μg/ml streptomycin, 100 U/ml penicillin, and 0.25 μg/ml fungizone (full growth medium). Cells used for experiments were at passages three to five.

Human dermal microvascular endothelial cells (HDMECs) were isolated in the laboratory of Dr. Peter Petzelbauer (Medical University of Vienna, Austria) from foreskins as previously described^68^. Cells were cultured in endothelial cell growth medium EGM-2 supplemented with EGM-2 BulletKit (LONZA) and FBS of final concentration 15% and used for experiments at passage five.

### Induction of senescence by chronic exposure to TNFα

For the induction of senescence, cells were seeded into 1% gelatin-coated 96 well plates in full growth medium and incubated for 24 h. Thereafter, cells were exposed to TNFα (10 ng/ml) alone or TNFα in combination with plumericin (1.5 μM), PHA-408 (2 μM) or NAC (2 mM) for the indicated time points. Fresh full growth medium containing TNFα (10 ng/ml) with or without inhibitors was supplied every other day, i.e. at day two and day four. On day six, cells were switched back into full growth medium without TNFα or inhibitors and kept cultivated for additional three to 10 days.

### Assessment of cellular proliferation of HUVECs by absolute cell count

The proliferation of HUVECs was assessed by absolute cell counting at the indicated time points. For this purpose, 1.5 × 10^3^ cells were seeded into 1% gelatin-coated 96-well plates and treated with TNFα (10 ng/ml) alone or in combination with plumericin (1.5 μM), PHA-408 (2 μM), and NAC (2 mM). The cells were fixed for 10 min in 4% PFA, washed with PBS and stained with Hoechst 33342 for nuclear staining at the indicated time points. Cells were imaged with the multiplex reader Cytation 5 and counted using Gene 5 software version 2.07, both from BioTek (Winooski, USA).

### Immunostaining for Ki-67, p21 and p16

HUVECs were exposed to TNFα (10 ng/ml) with or without plumericin, PHA-408, and NAC for the indicated time points. Cells were then fixed for 10 min in 4% PFA, washed with PBS, permeabilized with 0.2% Triton-X 100, blocked with 10% goat serum, and stained with primary antibody for proliferation marker Ki-67 or cyclin-dependent kinase inhibitors p21 and p16, followed by washing with PBS and staining with secondary antibody Alexa 555. Thirty fluorescent images of cells were taken randomly with the multiplex reader Cytation 5, following staining. The images were assessed using the software Gene 5 version 2.07.

### Assessment of apoptosis

Apoptosis was detected using a combination of propidium iodide staining and Annexin V-FITC Apoptosis Detection Kit (eBioscience, San Diego, CA, USA). In order to induce apoptosis as a positive control, HUVECs were treated with 500 μmol H_2_O_2_ for 90 min.

### Determination of NF-κB nuclear translocation in HUVECs

Nuclear localization of NF-κB subunit p65 was evaluated in HUVECs grown in 1% gelatin coated 96 well plates, in the presence or absence of TNFα (10 ng/ml), or in combination with plumericin (1.5 μM), PHA-408 (2 μM) or NAC (2 mM) for the indicated time points. Subsequently, the cell were fixed and stained with anti-p65 subunit of NF-κB, as previously described^69^. Fluorescent images were taken on an IX-50 Olympus microscope using a 10x lens and a cooled CCD camera, F-View II (Olympus, Münster, Germany). The mean integrated intensity of the p65 in nucleus was evaluated by using the cell image analysis software CellProfiler 2.1.

### Measurement of intracellular ROS in HUVECs

Intracellular ROS was measured in HUVECs as previously described^44^ by using the fluorescent probe H2-DCF with slight modifications. Briefly, HUVECs grown in 1% gelatin-coated 96-well plates overnight were treated either with TNFα (10 ng/ml) alone or in the presence of inhibitors for the indicated periods. At the end of the treatment period, cells were incubated at 37 °C with 10 μM H2-DCF in phenol red-free medium M199, supplemented with 10% FBS for 30 min in dark and the fluorescence was measured at an excitation wave length of 490 nm and an emission wavelength of 525 nm by a 96 well fluorescent plate reader Synergy 3 (BioTek).

### Detection of E-selectin and ICAM-1 by cell ELISA

Surface expression of E- selectin and ICAM-1 in HUVECs exposed to TNFα (10 ng/ml) or TNFα with NF-κB inhibitors plumericin (1.5 μM), PHA-408 (2 μM) or NAC (2 mM) for the indicated times was measured by cell ELISA as described before^41^.

### Detection of IL-6 and IL-8

Conditioned media were collected from HUVECs and HDMECs and analyzed for IL-6 and IL-8 using an ELISA kit (eBioscience, San Diego, CA, USA).

### QPCR analysis

Gene expression of IGFBP-5 and PAI-1 was determined by RNA isolation and subsequent qPCR. Shortly, total RNA from HUVECs grown in 6 well plates was isolated and reverse-transcribed. The following primers designed with the Primer 3 software were used: for IGFBP-5: 5′-GAG CTG AAG GCT GAA GCA GT-3′ (fwd.) and 5′-GAA TCC TTT GCG GTC ACA AT-3 (rev.) and for PAI-1: 5′-CAG ACC AAG AGC CTC TCC AC-3′ (fwd.) and 5′-ATC ACT TGG CCC ATG AAA AG-3′ (rev.). The expression levels of these genes were normalized to the expression of β2-microglobulin using 5′-GAT GAG TAT GCC TGC CGT GTG-3′ (fwd.) and 5′-CAA TCC AAA TGC GGC ATC T-3′ (rev.) primers.

### Statistical analysis

All experiments were performed at least in triplicate if not stated otherwise. Statistical analysis was carried out by using one way ANOVA/Newman Keuls post-test or unpaired two tailed t-test (when comparing just two experimental groups) with the help of GraphPad Prism version 5 (La Jolla, CA, USA). P values < 0.05 were considered as significant.

## Additional Information

**How to cite this article**: Khan, S. Y. *et al*. Premature senescence of endothelial cells upon chronic exposure to TNFα can be prevented by N-acetyl cysteine and plumericin. *Sci. Rep.* **7**, 39501; doi: 10.1038/srep39501 (2017).

**Publisher's note:** Springer Nature remains neutral with regard to jurisdictional claims in published maps and institutional affiliations.

## Supplementary Material

Supplementary Information
