# Supplementary material for: Premature senescence of endothelial cells upon chronic exposure to TNFα can be prevented by N-acetyl cysteine and plumericin
Source: Sci Rep. 2017 Jan 3;7:39501. doi: 10.1038/srep39501 (PMC5206708; doi:10.1038/srep39501)

Premature senescence of endothelial cells upon chronic exposure to TNFα can be prevented by N-acetyl cysteine and plumericin

Shafaat Y Khan[1,5], Ezzat M Awad[1], Andre Oszwald[1], Manuel Mayr[2], Xiaoke Yin[2], Birgit Waltenberger[3], Hermann Stuppner[3], Markus Lipovac[4], Pavel Uhrin[1] and Johannes M. Breuss[1,*]

[1]Department of Vascular Biology and Thrombosis Research, Center for Physiology and Pharmacology, Medical University of Vienna, 1090 Vienna, Austria.

[2]King's British Heart Foundation Centre, King's College London, London SE5 9NU, UK.

[3]Institute of Pharmacy/Pharmacognosy and Center for Molecular Biosciences Innsbruck (CMBI), University of Innsbruck, 6020 Innsbruck, Austria.

[4]Karl Landsteiner Institute for Cell-based Therapy in Gynecology, 2100 Korneuburg, Austria.

[5]Department of Zoology, University of Sargodha, 40100 Sargodha Pakistan.

[*]Email: Johannes.Breuss@meduniwien.ac.at

Supplemental Information

Supplemental Figure 1. Characterization of HDMECs. (**a**) Increased portion of p16 positive cells and (**b**) an increase in size and pronounced cell flattening after six days of TNFα (10ng) treatment. (**c**) Increased IL-6 and (**d**) IL-8 levels after six days of TNFα treatment. Values are presented as mean ± SD . (***p<0.001). The results shown are derived from a single experiment in technical triplicates (**a**) or in duplicates (**c,d**).

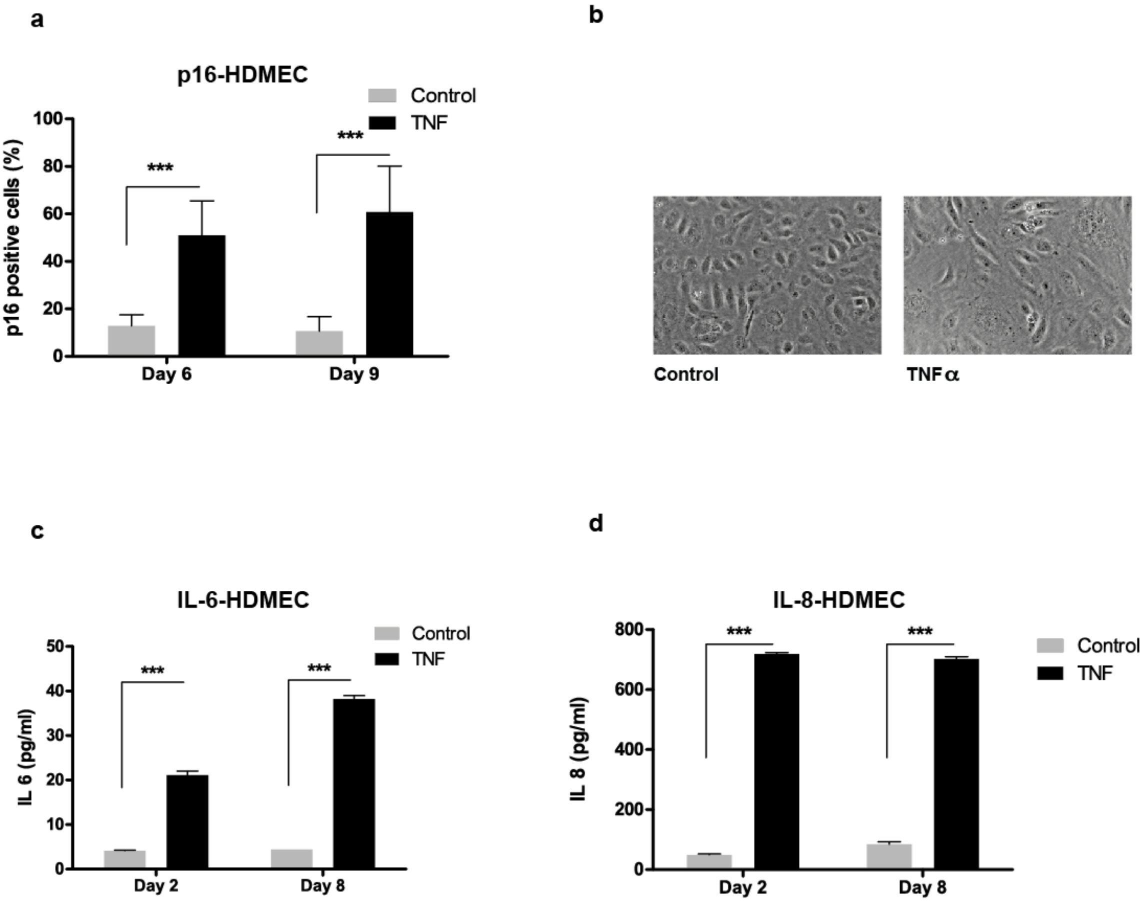

Supplemental Figure 2. Effects of cultivation of HUVECs in the presence of TNFα for six days, followed by an additional 7-10 days period without TNFα. (**a**) Retardation of growth in TNFα (10ng) treated HUVECs. (**b**) Increased portion of p16 positive cells after TNFα treatment, as assessed at days 13 and 16. Increased levels of (**c**) IL-6 and (**d**) IL-8 throughout the entire analyzed period. (**e**) Elevated ROS production in TNFα treated cells at days 8, 10 and 13 as compared to controls cells. (**f**) Quantification of apoptosis as determined by annexin V and propidium iodide staining and subsequent evaluating of at least 300 cells. H_2O_2 was used as positive control for the induction of apoptosis. Values are presented as mean ± SD of a single experiment in technical triplicates (**a,b,e,f**) or duplicates (**c,d**). (***p<0.001).

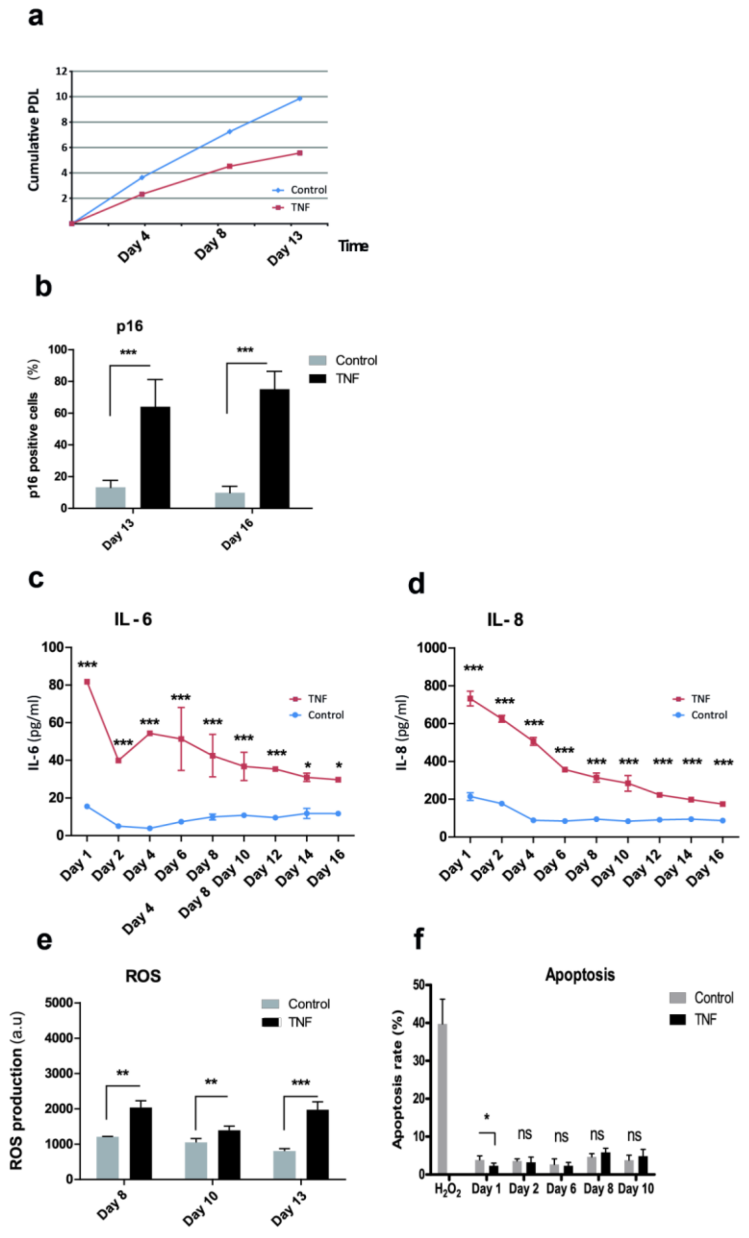

Supplemental Figure 3. Effects of plumericin, PHA-408, and NAC on growth rates and cytokine secretion in HUVECs. (**a**) Growth curves of HUVECs treated with inhibitors only. The results shown are representative of three independent experiments accomplished in technical triplicates. Levels of (**b**) IL-6 and (**c**) IL-8 in HUVECs treated with inhibitors only. The results shown are derived from a single experiment in technical duplicates. PL: plumericin, PHA: PHA-408, NAC: N-acetyl cysteine.

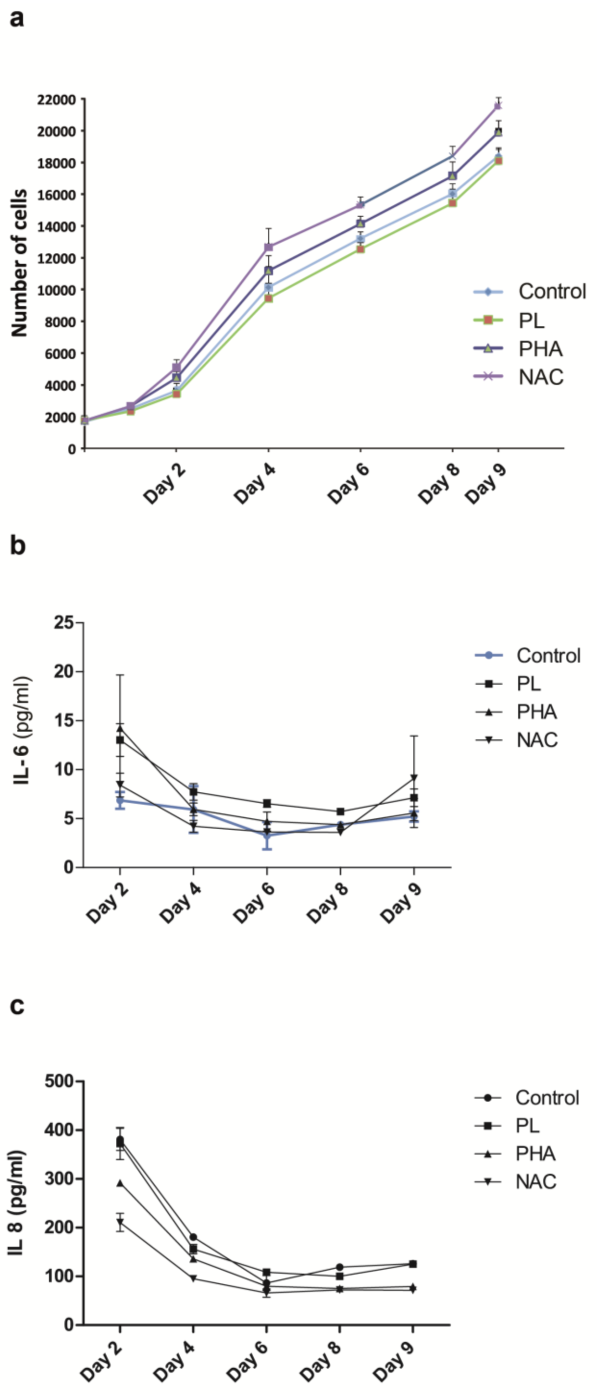

Supplement: Supplementary Information [file srep39501-s1.pdf]
